# Environmentally sustainable synthesis of whey-based carbon dots for ferric ion detection in human serum and water samples: evaluating the greenness of the method

**DOI:** 10.1039/d3ra08680a

**Published:** 2024-02-08

**Authors:** Kawan F. Kayani, Omer B. A. Shatery, Muhammad S. Mustafa, Azad H. Alshatteri, Sewara J. Mohammed, Shujahadeen B. Aziz

**Affiliations:** a Department of Chemistry, College of Science, University of Sulaimani Qliasan St 46002 Sulaimani City Kurdistan Region Iraq Sewara.mohammed@univsul.edu.iq; b Department of Chemistry, College of Science, Charmo University Peshawa Street, Chamchamal Sulaimani City 46023 Iraq; c Department of Chemistry, College of Education, University of Garmian Kalar 46021 Sulaimani Kurdistan Region Iraq; d Anesthesia Department, College of Health Sciences, Cihan University Sulaimaniya Sulaimaniya 46001 Kurdistan Region Iraq; e Hameed Majid Advanced Polymeric Materials Research Lab., Research and Development Center, University of Sulaimani Qlyasan Street Sulaymaniyah Kurdistan Region 46001 Iraq; f Department of Physics, College of Science, Charmo University Chamchamal 46023 Sulaymaniyah Iraq

## Abstract

Carbon dots (CDs) are valued for their biocompatibility, easy fabrication, and distinct optical characteristics. The current study examines using whey to fabricate CDs using the hydrothermal method. When stimulated at 350 nm, the synthetic CDs emitted blue light at 423 nm and revealed a selective response to ferric ion (Fe^3+^) in actual samples with great sensitivity, making them a suitable probe for assessing Fe^3+^ ions. The produced carbon dots demonstrated great photostability, high sensitivity, and outstanding biocompatibility. The findings showed that Fe^3+^ ions could be quickly, sensitively, and extremely selectively detected in an aqueous solution of carbon dots, with a revealing limit of 0.409 μM in the linear range of 0–180 μM. Interestingly, this recognition boundary is far inferior to the WHO-recommended threshold of 0.77 μM. Two metric tools which were AGREE and the ComplexGAPI were also used to evaluate the method's greenness. The evaluation confirmed its superior environmental friendliness.

## Abstract 2

Carbon dots (CDs) are valued for their biocompatibility, easy fabrication, and distinct optical characteristics.

## Graphical abstract

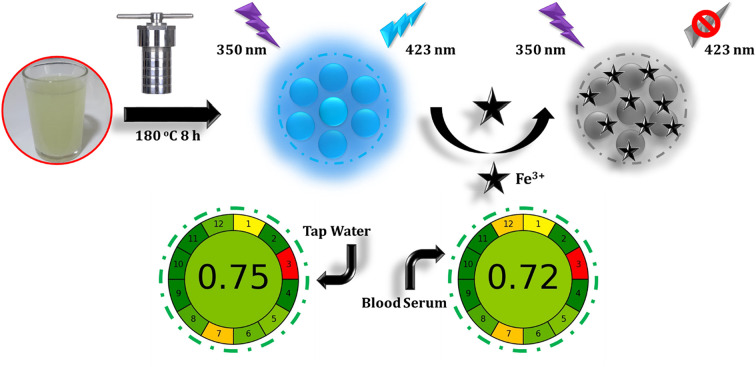

## 1 Introduction

CDs, or luminescent nanoparticles, have garnered widespread attention in recent years. They demonstrate fluorescence when exposed to ultraviolet or visible light.^1–4^ Moreover, they possess little toxicity,^5^ outstanding photostability,^6^ and high water solubility.^7^ There are two main synthetic approaches for CDs: the top-down and bottom-up methods, which are categorized based on the relationship between sources and products.^8^ The top-down method involves the utilization of carbon-rich materials or molecules as carbon sources, cutting larger carbon structures like graphite, carbon nanotubes, carbon soot, activated carbon, and graphite oxide into smaller CDs through techniques such as arc discharge,^9^ laser ablation^10^ and electrochemical methods.^11^ On the other hand, bottom-up methods use organic molecules, such as glucose^12^ and fructose^13^ to react and form larger CDs by applying external energy like ultrasonication,^14^ microwave pyrolysis,^15^ heating,^16^ hydrothermal,^17^ and microwave irradiation.^18^ Due to their unique properties, CDs have found applications in various fields, replacing toxic semiconductor quantum dots^19^ made of heavy metals like cadmium^20^ and lead.^21^ CDs are used in sensing,^22^ electronics,^23^ photocatalysis,^24^ and biomedical applications.^25,26^ They can be synthesized from diverse precursors, including wastes, edibles, and chemical substances,^27^ such as watermelon peel,^28^ mango,^29^ garlic,^30^ and ethanol.^31^ CDs have been applied in imaging mycobacterium and fungal cells, using carbon dots derived from apple juice,^32^ and in drug delivery and gene transfer.^33^ Additionally, nitrogen-doped carbon dots demonstrated by Liang served as an “on–off–on” fluorescent sensor for Fe^3+^ and glutathione detection.^34^

In this study, we developed a method for synthesizing CDs from yogurt, a widely popular fermented food enjoyed globally. Renowned for its delightful taste, yogurt frequently features on lists of healthy foods due to its rich content of vitamins, minerals, and proteins, a known source of nitrogen atoms that enhance the quantum efficiency of CDs.^35^ Moreover, yogurt is affordable and widely accessible in most supermarkets and convenience stores. This marks the pioneering synthesis of CDs from fermented milk water using the hydrothermal method. The characterization of the CDs was conducted with great care using UV-vis, fluorescence, FTIR, XRD spectroscopy, DLS, and TEM.

These CDs found application in various sensing scenarios, particularly in the detection of the metal ion Fe^3+^ in real samples. Our developed fluorescence sensor demonstrated excellent accuracy and stability in determining Fe^3+^ levels in drinking water and blood samples. Furthermore, CDs exhibited a selective response to Fe^3+^ even in complex biological environments.

Metal ions play a crucial role in regulating the health of the human body, influencing various biological processes, and serving as essential elements for enzymatic reactions.^36,37^ Among these, iron stands out as a vital metal ion, contributing to normal biological functions alongside other essential ions like sodium, calcium, zinc, magnesium, and cobalt. Notably, iron functions as a central component in proteins such as hemoglobin, the blood protein.^38^ Due to its involvement in electron transfer and essential biological processes like cellular respiration, energy production, and oxygen transport,^39–41^ iron plays a pivotal role in enzymatic reactions. However, an imbalance either excess or deficiency, can lead to molecular diseases and blood disorders, including hemochromatosis, anemia, decreased immunity, and low blood pressure.^42–45^ Maintaining the proper concentration of iron is crucial for sustaining the body's functions.

Water, a fundamental resource for human and environmental well-being, faces significant threats from pollution. Major contributors to water pollution include the discharge of domestic sewage, industrial wastes (from sectors like textile, leather, and dyeing), radioactive wastes, as well as fertilizers and pesticides from agricultural fields. The accumulation of industrial wastes and heavy metals in water bodies poses serious threats to both human and animal life.^46^ Therefore, there's a need for simple, effective, and eco-friendly sensor designs for metal ion detection.

Detecting metal ions at low concentrations poses a considerable challenge. Traditional methods like Atomic Absorption Spectroscopy (AAS),^47,48^ Inductively Coupled Plasma-Mass Spectrometry (ICP-MS),^49^ and electrochemical assays like voltammetry,^50^ are not only time-consuming but also demand expensive equipment and meticulous sample preparation. Consequently, straightforward fluorescence probes have surfaced as encouraging sensors. Their attributes, such as high quantum yield, ease of modification, excellent biocompatibility, heightened sensitivity, and the ability to provide real-time sensing.^51,52^ These advantages make them valuable tools in addressing the challenges associated with metal ion detection.^53–55^

In this study, we used whey extracted from yogurt as the main material for synthesizing carbon quantum dots (CDs) with a distinct fluorescent emission peak at 423 nm. The fluorescence at 423 nm, displayed by the CDs, can be quenched by Fe^3+^ ions. Leveraging the strong binding affinity between CDs and Fe^3+^ ions, we can explore the application of these CDs for detecting Fe^3+^ ions in both serum and tap water.

## 2 Experimental details

### 2.1 Materials

The yogurt used in this study was purchased from a local supermarket in Chamchamal, Iraq. All the chemicals available in the laboratory were utilized. The metal ions had the following purities: Fe(NO_3_)_3_·9H_2_O, ≥99%, Al(NO_3_)_3_·9H_2_O ≥ 99%, Co(NO_3_)_2_·6H_2_O ≥ 98%, Cu(NO_3_)_2_·3H_2_O ≥ 99%, Hg(NO_3_)_2_·H_2_O ≥ 98%, Mn(NO_3_)_2_·4H_2_O ≥ 97%, Mg(NO_3_)_2_·6H_2_O ≥ 98%, Ni(NO_3_)_2_·6H_2_O ≥ 98.5%, Zn(NO_3_)_2_·6H_2_O ≥ 98%, Pb(NO_3_)_2_ ≥ 99%, Cd(NO_3_)_2_·4H_2_O 98%. The molecules had the following purities: citric acid (CA, ≥99.5%), arginine (Arg, ≥98%), alanine (Ala, ≥98.5%), l-aspartic acid (l-AA, ≥98%), glycine (Gly, ≥98.5%), ascorbic acid (AA, 99%), histidine (His, ≥99%), phenylalanine (Phy, ≥98%), and cysteine (Cys, ≥97%). Deionized (DI) water was employed in all experiments. Deionized (DI) water with a resistivity of 10 MΩ cm and a conductivity of 0.1 μS was used in all experiments.

### 2.2 Apparatus

A Cary 60 spectrophotometer from Agilent Technologies, USA, was used to obtain the UV-vis absorbance spectrum (200–600 nm), while a Cary Eclipse Fluorescence spectrophotometer, also from Agilent Technologies, USA, was employed for spectrofluorometric scanning. The FEI Tecnai G2 F30 was used for High-Resolution Transmission Electron Microscopy (HRTEM), and a Nicolet FTIR spectrometer from Thermo Scientific, USA, was utilized. X-ray diffraction (XRD) analysis was conducted using an Empyrean XRD with a Cu Kα radiation source.

### 2.3 Synthesis of carbon dots

**Scheme 1:**
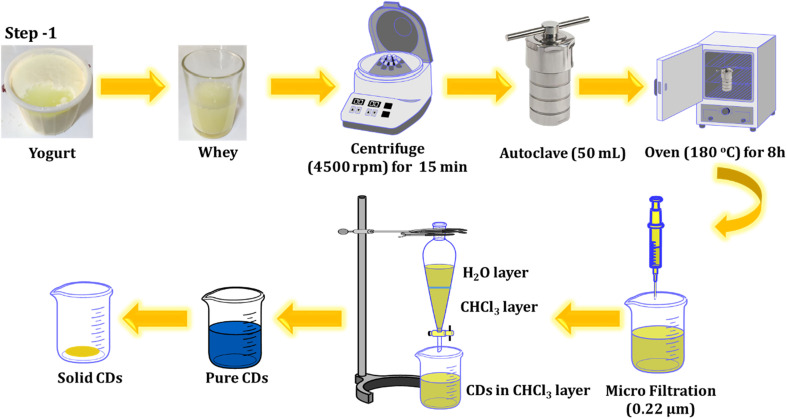
Illustration of the process for synthesizing the carbon material.

Yogurt was obtained after purchasing at a local supermarket in Chamchamal, Iraq. The whey was then collected from the yogurt and utilized for the fabrication of C-dots through a one-step hydrothermal process. The solution was centrifuged at 4500 rpm for 15 minutes to remove impurities, and the resulting supernatant was poured into a Teflon-lined stainless-steel chamber. It was then heated at 180 °C for 8 hours in an oven. The resultant solution underwent another round of centrifugation (12 000 rpm for 10 minutes) and was filtered through a sterile PTFE syringe filter (0.22 μm). To eliminate any residual unreacted species molecules, an extraction process was conducted twice, using chloroform as a solvent. The product was collected and refrigerated for further experiments. The process of preparing the luminescent CDs from whey is outlined below and illustrated in Scheme 1.

### 2.4 Analytical application

For the detection of Fe^3+^ ions, 0.5 mL of purified CD solution was mixed with varying amounts of ferric nitrate solution. The mixture was then diluted with deionized water to a total volume of 4 mL. Subsequently, the fluorescence spectra of the solution were recorded and utilized for quantitative analysis.

### 2.5 Detection of Fe^3+^ in serum and tap water

The initial step involved treating human serum to liberate Fe^3+^ from proteins. Essentially, equal amounts of human serum and pure ethanol were combined and heated at 95 °C for 15 minutes. Following cooling to room temperature, the serum solution underwent sonication for 2 minutes. Subsequently, the centrifugation process was employed to eliminate the protein precipitate, and the resulting supernatant was gathered for subsequent use.^56^ All experiments involving human blood serum samples were performed in compliance with the relevant laws and guidelines of Iraq. The experiments followed the institutional guidelines of Chamchamal Sub DOH Central Public Health Laboratory and were approved by its Ethical Committee (Approval No. CPHC100). Informed consent was obtained from all human subjects who participated in the study. In assessing the practicality of the carbon dots-based sensor for detecting Fe^3+^ in authentic samples, tap water samples sourced from our laboratory were subjected to analysis using the described method. All the samples of the water were spiked with Fe^3+^ at varying concentrations without undergoing any pre-treatment.

## 3 Results and discussion

### 3.1 Characterization

**Fig. 1:**
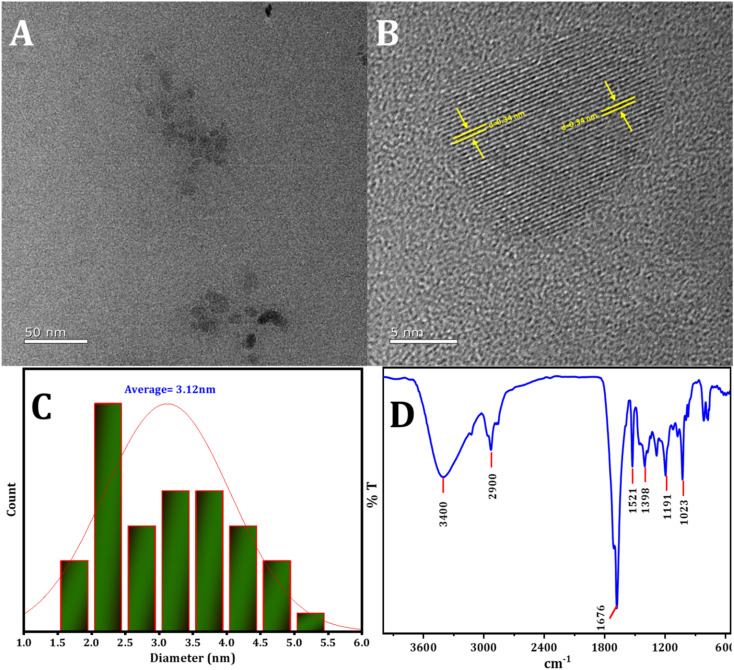
(A) and (B) The HRTEM image of CDs, (C) the statistical distribution of particle sizes of CDs, (D) the pattern of FTIR spectra of CDs.

The structure and dimensions of the synthesized CDs were examined using HRTEM, as depicted in Fig. 1. Fig. 1A and B illustrate that the CDs exhibit uniform spherical shapes and are evenly dispersed. The size distribution, presented in the histogram (Fig. 1C), indicates a range from 1 to 5 nm, with a mean particle diameter of roughly 3.12 nm. Furthermore, the lattice fringes observed in the HRTEM image (Fig. 1C) have a *d*-spacing of approximately 0.34 nm, consistent with the lattice of graphitic carbon's (100) plane.^57,58^ Interestingly, the CDs do not exhibit a distinct crystal lattice, implying an amorphous structural nature.

FTIR examination was conducted to investigate the active groups present on the surface of the CDs. In Fig. 1B, the following observations were made: stretching vibrations of O–H and N–H in the region 2900 cm^−1^ to 3400 cm^−1^,^59^ an absorption peak at 1676 cm^−1^ corresponding to the stretching vibration of C=O,^60^ the absorption peak at 1521 cm^−1^ attributed to the stretching vibration of C=C, and the peak at 1398 cm^−1^ identified as C–N, N–H. Additionally, the peak at 1191 cm^−1^ was ascribed to the C–O, C–N, and C–S bonds.^61^ The peak at 1023 cm^−1^ is assigned to the stretching vibration of S=O.^62^

**Fig. 2:**
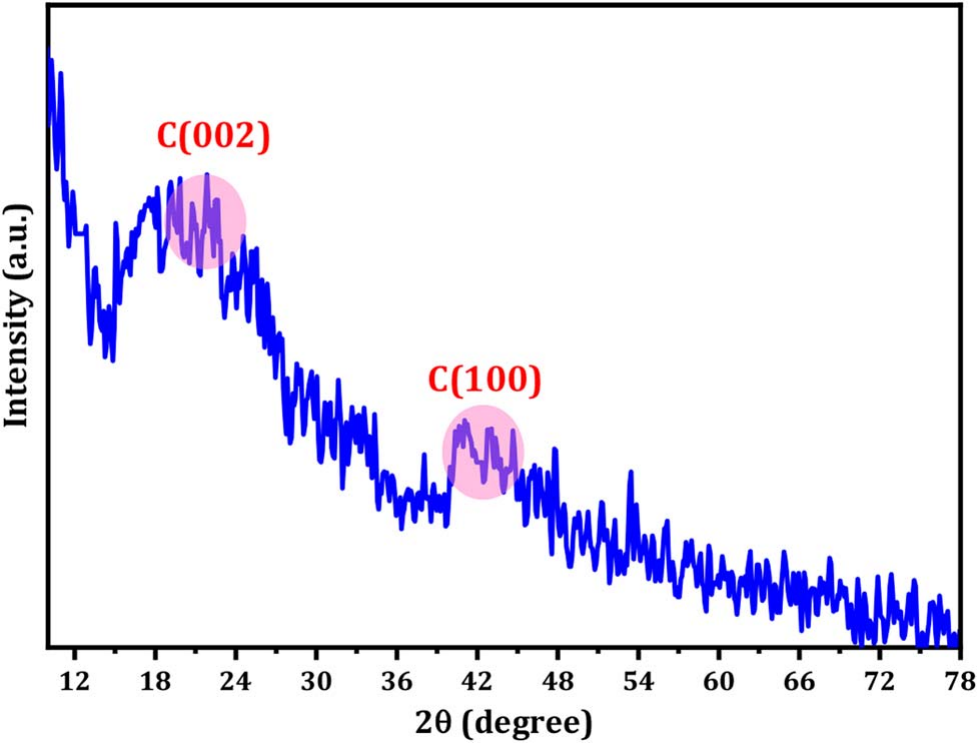
The XRD spectra of the prepared CDs.

The XRD pattern of the CDs displays a range of broad peaks, indicating the presence of highly disordered carbon atoms in the as-prepared CDs (refer to Fig. 2).^63,64^ The CDs exhibited broad 2*θ* patterns around 22.5 and 41°, attributed to disordered carbon atoms which confirms the dominance of C(002) and C(100) planes associated with hexagonal graphite structure of CDs particles.^65^ The broad XRD pattern is evidence for the crystalline particles with small dimensions. The particle size distribution (Fig. 1C) supports the XRD approach. The Debye–Scherrer equation (*D* = *Kλ*/*β* cos *θ*) establishes the fact that the produced CDs are of small size ascribing to the fact the *β*-factor in the denominator of the equation is too high for broad peak around 2*θ* = 15–30° and consequently the crystallite size will be very small.

### 3.2 The FL properties of CDs

**Fig. 3:**
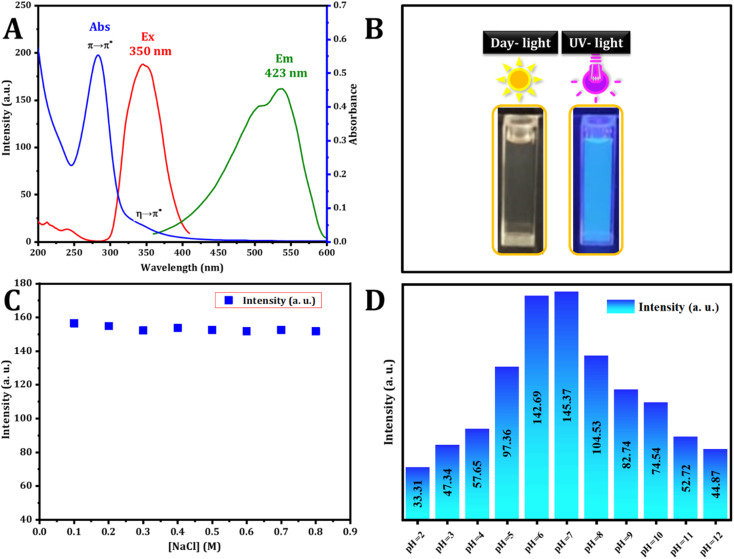
(A) Absorption (blue line), excitation (red line), and emission (green line) spectra of CDs. (B) Photographs depicting these carbon dots under daylight (left) and 365 nm UV light (right) illumination. (C) Examination of ionic strength at various concentrations of NaCl (0.1 to 0.8 M). (D) pH study of CDs (pH = 2.0–12.0).

The optical characteristics of the carbon dots were assessed using UV-vis and fluorescence spectroscopy. The UV-vis spectrum, illustrated in Fig. 3, revealed a wide absorption band spanning from 250 to 420 nm. Notably, the absorption bands peaked at 283 and 347 nm, matching the π–π* transition and the n–π* transition ascribed to aromatic sp^2^ hybridization and C=O bonds respectively. These peaks are attributed to the existence of carbonyl-based groups on the surface.^35^ The excitation and emission spectra were analyzed, and the excitation spectra were obtained by monitoring the emission at 423 nm, revealing a strong peak at 350 nm. The dots exhibited blue emission at 423 nm under 350 nm excitation. The visual confirmation of C-dot synthesis was established. As depicted in Fig. 3B, hydrothermal synthesis produces a colorless solution (in daylight) that transforms into a blue color under UV excitation (360 nm) of the aromatic sp^2^ domains.

### 3.3 The stability experiment

The durability of photoluminescence exhibited by CDs in aqueous settings is a subject of significant attention for realistic claims. Various factors, such as pH and ionic strength, can influence their recital. To explore this, the study initially examined the fluorescence emission intensity of CDs in solutions containing salt and in different aqueous environments at varying pH levels. The strength of the emission experienced minimal impact across various concentrations of NaCl solutions (Fig. 3C), aligning with findings from a prior study. Fig. 3D illustrates how the fluorescence intensity of CDs is influenced by pH. The CDs synthesized exhibit stability within the pH range of 2.0 to 12.0, rendering them suitable for a variety of applications.

**Fig. 4:**
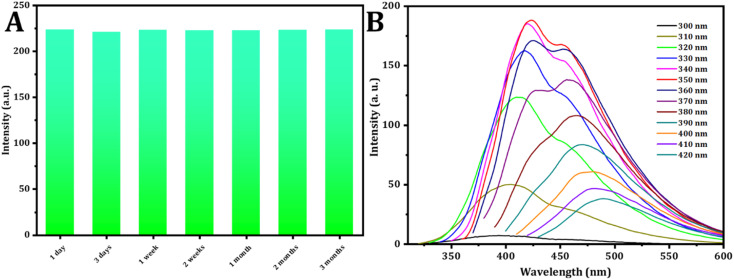
(A) Effect of time on the intensity of the CDs. (B) Study of excitation spectra (from 300 to 420 nm) of CDs.

The stability analysis of CDs reveals no significant change in fluorescence intensity during the three month storage period, as depicted in Fig. 4A. This result suggests that the CDs remained stable even after three months, with minimal variation.

The fluorescence spectra of the C-dots solution, prepared under normal temperature conditions, were recorded. When excited at varying wavelengths spanning from 300 nm to 420 nm with a 10 nm increment, as shown in Fig. 4B, the FL intensity of emission peaks initially increased under excitation from 300 to 350 nm. Subsequently, after excitation at 350 nm, it gradually decreased. These findings indicate that the optimal emission was observed at 423 nm when excited at 350 nm.

### 3.4 Selectivity studies

**Fig. 5:**
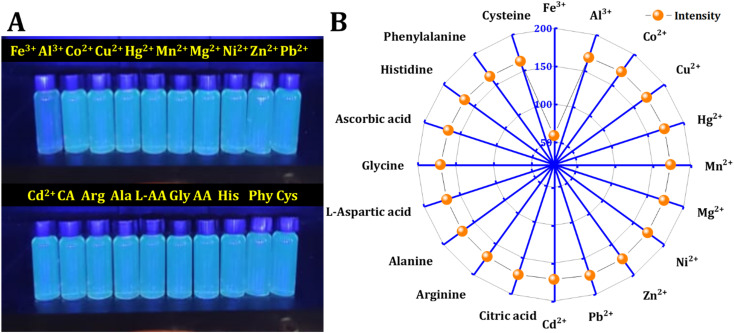
Interferences: (A) naked-eye detection of metal ions and molecules. (B) Fluorescence intensity of CD in the presence of 500 μM of various metal ions and molecules.

Various metal ions and molecules may exist in the real solution. The influence of 20 kinds of metal ions and molecules on the fluorescence intensity is compared. The procedure involved creating stock solutions of metals and molecules (0.01 M) and adding 500 μL of the probe to a total volume of 4 mL, resulting in a final concentration of interferences (1 mM). The fluorescence intensity of the mixtures was then recorded at an excitation wavelength of 350 nm. It was found that only Fe^3+^ ions can selectively quench the fluorescence of synthetic materials, as shown in Fig. 5. Metal ions, including Fe^3+^, Al^3+^, Co^2+^, Cu^2+^, Hg^2+^, Mn^2+^, Mg^2+^, Ni^2+^, Zn^2+^, Pb^2+^, and Cd^2+^, along with molecules such as citric acid (CA), arginine (Arg), alanine (Ala), l-aspartic acid (l-AA), glycine (Gly), ascorbic acid (AA), histidine (His), phenylalanine (Phy), and cysteine (Cys), were chosen to investigate their influence on C-dots.

### 3.5 Analytical application

**Fig. 6:**
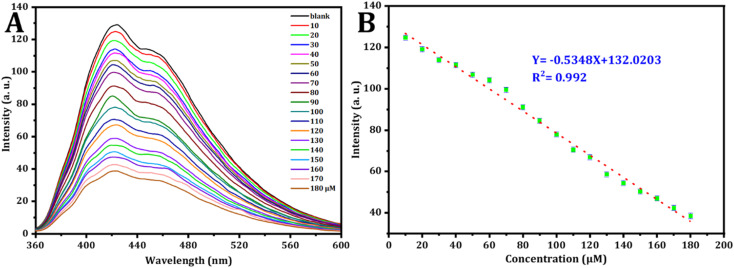
(A) The fluorescence behavior of CDs in the existence of varying concentrations of Fe^3+^ was examined. (B) Corresponding relationship between FL intensity and the concentrations of Fe^3+^ in μM.

**Fig. 7:**
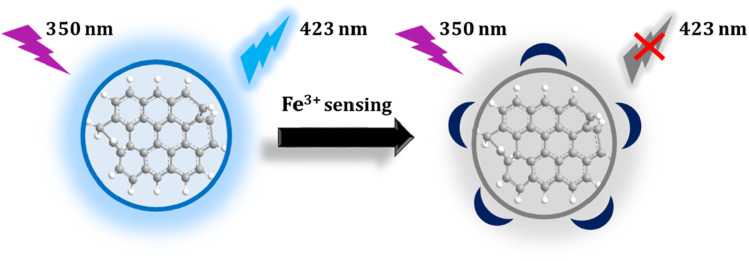
Schematic of the CDs as an on–off' fluorescent sensor for the detection of Fe^3+^.

To conduct a quantitative investigation and set up calibration curves, a series of standard Fe^3+^ solutions were examined. Following optimized circumstances; the calibration plot was generated by drawing fluorescence intensity against concentrations of standard Fe^3+^, which is determined by the different degrees of the inter-filter effect, as depicted in Fig. 7.^66^ As seen in Fig. 6A, the fluorescence intensity exhibited a decrease as the Fe^3+^ concentration increased from 10 μM to 180 μM. simultaneously, the fluorescence emission peak at 423 nm lowered. Fig. 6B describes the relationship between Fe^3+^ ion concentration and FL intensity in the range from 10 to 180 μM, in accordance with the following equation:*F*_423_ = 132.0203 − 0.5348[Fe^3+^]

The correlation coefficient is 0.992, the calculated detection limit for Fe^3+^ ions is 0.409 μM (S/N = 3) and the limit of quantification is 1.365 μM.

CDs have high sensitivity compared with different probes that have been used to quantification of Fe^3+^ in drinking water as shown in Table 1.

**Table 1:** Comparison of other CDs as probes for fluorescence Fe^3+^ detection in real samples

Probe	Real sample	Linear range (μM)	LOD	Ref.
N,S–GQDs	Drinking water	0.1–30	55.49 nM	67
CDs	Drinking water	8–80	3.8 μM	68
CDs	Tap water	0.3–7	95 nM	69
CDs	Lake water	0.0–250	1.68 μM	70
N–CQDs/PTCA	Tape water	20–60	0.04 μM	71
CDs	Milk	0.0–200	35 nM	72
Phe–CDs	Tap water	5.0–500	0.720 μM	73
CDs	Underground water	30–600	9.55 μM	74
N–CQDs	Tap water	0.5–1000	0.079 μM	75
CDs	Serum and tap water	0–180	409 nM	This work

### 3.6 Detection of Fe^3+^ in real samples

To assess the applicability of this developed technique, standard recovery experiments were carried out using samples of human serum and tap water (refer to Table 2). The results in Table 2 indicate that the recoveries for all samples ranged from 95.60% to 102.46% for human blood serum and from 83.75% to 90.26% for tap water. The associated relative standard deviations (RSDs) were within the ranges of 0.96% to 2.66%. These findings provide robust evidence supporting the potential of the proposed approach for the practical detection of Fe^3+^ in actual samples.

**Table 2:** Recovery results and spiking recoveries of Fe^3+^ in human blood serum, and water (*n* = 3)

Sample	Added (μM)	Found (μM)	Recovery%	RSD%
Serum	80	77.31	95.60	1.87
	90	95.74	102.46	2.08
Tap water	40	36.22	83.75	0.96
	50	47.85	90.26	2.66

### 3.7 Greenness profile of the method

Recently, energy, waste, and hazard management have been recognized as crucial to improving green analysis. In this work, three different green metrics were utilized to evaluate the greenness of the proposed approach, comprising the analytical eco-scale (AES),^76^ the complimentary green analytical procedure index (ComplexGAPI),^77^ and the Analytical Greenness approach (AGREE).^78^ AES is a simple and semi-quantitative tool that is based on penalty points (PP) for different experimental steps subtracted from a total score of 100. The PPs are assigned for each step of analytical parameters such as the nature and quantity of solvents and reagents employed, energy consumption, hazards, and waste generated. From the results listed in Table 3, one can observe that the proposed method was given a score of 82 for tap water and 78 for blood serum samples, which confirms the greenness of the approach.

**Table 3:** Greenness evaluation of the proposed method according to the AES^a^

Category	PPs (tap water)	Category	PPs (blood serum)
**Reagents**			
Water	0	Water	0
Ferric nitrate	6	Ferric nitrate	6
Chloroform	6	Chloroform	6
		Ethanol	4

**Instruments**			
Spectrofluorometer	0	Spectrofluorometer	0
Occupational hazard	0	Occupational hazard	0
Waste	6	Waste	6
Total PPs: 18 score: 82		Total PPs: 22 score: 78	

a PPs: penalty points. The assessment was conducted on a per-sample basis. Scores exceeding 75 are classified as excellent green analysis, scores within the range of 50–75 are considered acceptable green analysis, and scores below 50 are deemed inadequate green analysis.

The ComplexGAPI metrics software is an innovative tool built upon GAPI, featuring an additional hexagonal area at its base. ComplexGAPI is essential for encompassing the entire experimental process, including pre-analysis procedures spanning from sample preparation to the final analysis. It covers various aspects such as yield and environmental factors, association with the green economy, workup, instrumentation, reagents and solvents, and purification. As in GAPI, the color scale of the pictogram changes from green to yellow to red with respect to the low, medium, and high environmental effects. However, the overall results of ComplexGAPI, as shown in Fig. 7, indicate the greenness of the method. In the suggested procedure, micro-scale extraction was applied as a pre-treatment step for protein precipitation in the blood serum samples to improve the sensitivity of the analysis. Due to the minimal quantity of reagent and solvent waste generated, the procedure seems to be eco-friendly. Whey was utilized as an environmentally friendly carbon source in CD production. Furthermore, the spectrofluorimeter consumes little energy per analysis.

**Fig. 8:**
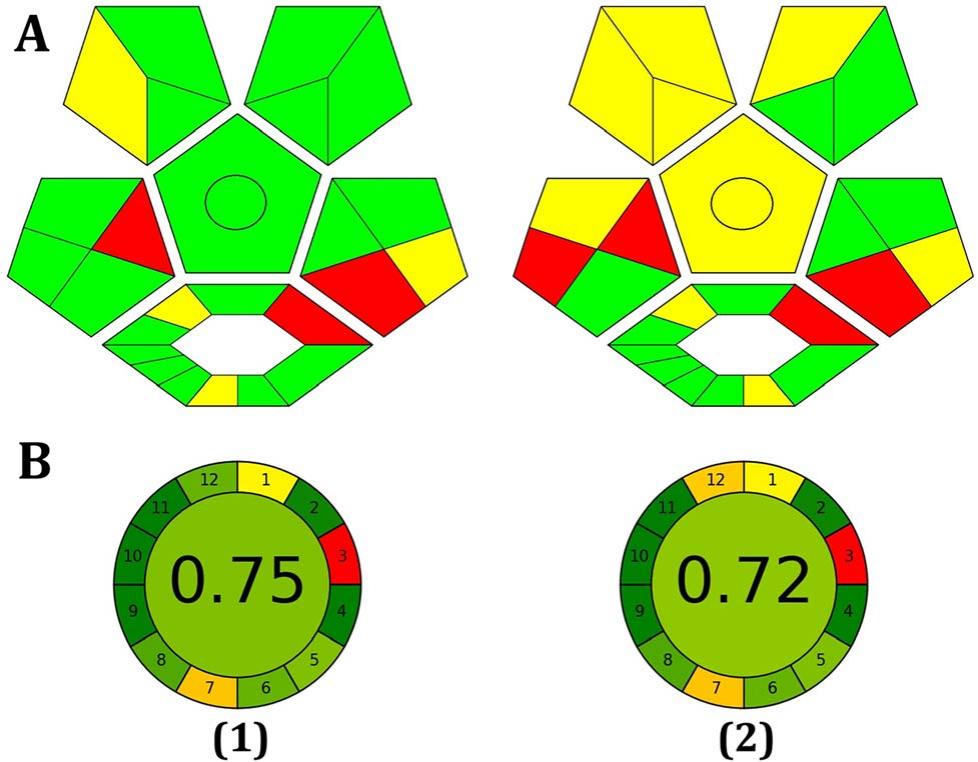
Evaluation of the environmental friendliness of the proposed method using ComplexGAPI (A) and AGREE (B) scales in tap water (1) and blood serum samples (2).

AGREE Metrics is a software application that depends on a set of 12 criteria to evaluate the environmental and occupational risks involved in an analytical procedure. These criteria are arranged in a circular pattern resembling the face of a traditional clock, with scores ranging from 0 to 1. The average score, situated at the center, serves as an indicator of the environmental sustainability of the approach. As illustrated in Fig. 8, the high score obtained with the AGREE software reflects the adoption of the excellent green methodology.

## 4 Conclusion

Using yogurt whey as a precursor, carbon dots were produced in a one-step hydrothermal method. When stimulated with the maximal excitation wavelength of 350 nm, the produced CDs emit blue fluorescent colors at 423 nm. The analytical figure of merit of CDs was investigated, and it demonstrates great selectivity and sensitivity with a low limit of detection for detecting Fe^3+^ ions in various matrixes. The addition of ferric ions may nearly completely suppress the fluorescence of carbon dots *via* a fluorescence “on/off” process. Furthermore, by analyzing multiple criteria for the method's greenness, we propose that these carbon dots, due to their greater environmental friendliness, might be used to monitor Fe^3+^ ions in a range of biological settings and for environmental monitoring.

## Ethical statement

This article does not contain any studies with human or animal subjects.

## Consent to participate

All authors provided their consent for the inclusion of their work in the manuscript.

## Data availability

All data generated and analyzed during the current study are included in the manuscript.

## Conflicts of interest

The authors declare no competing interests.

## Supplementary Material
